# Differences in Reporting of Analyses in Internal Company Documents Versus Published Trial Reports: Comparisons in Industry-Sponsored Trials in Off-Label Uses of Gabapentin

**DOI:** 10.1371/journal.pmed.1001378

**Published:** 2013-01-29

**Authors:** S. Swaroop Vedula, Tianjing Li, Kay Dickersin

**Affiliations:** Department of Epidemiology, Johns Hopkins Bloomberg School of Public Health, Baltimore, Maryland, United States of America; Harvard University, Brigham and Women's Hospital, United States of America

## Abstract

**Background:**

Details about the type of analysis (e.g., intent to treat [ITT]) and definitions (i.e., criteria for including participants in the analysis) are necessary for interpreting a clinical trial's findings. Our objective was to compare the description of types of analyses and criteria for including participants in the publication (i.e., what was reported) with descriptions in the corresponding internal company documents (i.e., what was planned and what was done). Trials were for off-label uses of gabapentin sponsored by Pfizer and Parke-Davis, and documents were obtained through litigation.

**Methods and Findings:**

For each trial, we compared internal company documents (protocols, statistical analysis plans, and research reports, all unpublished), with publications. One author extracted data and another verified, with a third person verifying discordant items and a sample of the rest. Extracted data included the number of participants randomized and analyzed for efficacy, and types of analyses for efficacy and safety and their definitions (i.e., criteria for including participants in each type of analysis). We identified 21 trials, 11 of which were published randomized controlled trials, and that provided the documents needed for planned comparisons. For three trials, there was disagreement on the number of randomized participants between the research report and publication. Seven types of efficacy analyses were described in the protocols, statistical analysis plans, and publications, including ITT and six others. The protocol or publication described ITT using six different definitions, resulting in frequent disagreements between the two documents (i.e., different numbers of participants were included in the analyses).

**Conclusions:**

Descriptions of analyses conducted did not agree between internal company documents and what was publicly reported. Internal company documents provide extensive documentation of methods planned and used, and trial findings, and should be publicly accessible. Reporting standards for randomized controlled trials should recommend transparent descriptions and definitions of analyses performed and which study participants are excluded.

*Please see later in the article for the Editors' Summary*

## Abstract 2

Using documents obtained through litigation, S. Swaroop Vedula and colleagues compared internal company documents regarding industry-sponsored trials of off-label uses of gabapentin with the published trial reports and find discrepancies in reporting of analyses.

## Editors' Summary

**Background:**

To be credible, published research must present an unbiased, transparent, and accurate description of the study methods and findings so that readers can assess all relevant information to make informed decisions about the impact of any conclusions. Therefore, research publications should conform to universally adopted guidelines and checklists. Studies to establish whether a treatment is effective, termed randomized controlled trials (RCTs), are checked against a comprehensive set of guidelines: The robustness of trial protocols are measured through the Standard Protocol Items for Randomized Trials (SPIRIT), and the Consolidated Standards of Reporting Trials (CONSORT) statement (which was constructed and agreed by a meeting of journal editors in 1996, and has been updated over the years) includes a 25-point checklist that covers all of the key points in reporting RCTs.

**Why Was This Study Done?:**

Although the CONSORT statement has helped improve transparency in the reporting of the methods and findings from RCTs, the statement does not define how certain types of analyses should be conducted and which patients should be included in the analyses, for example, in an intention-to-treat analysis (in which all participants are included in the data analysis of the group to which they were assigned, whether or not they completed the intervention given to the group). So in this study, the researchers used internal company documents released in the course of litigation against the pharmaceutical company Pfizer regarding the drug gabapentin, to compare between the internal and published reports the reporting of the numbers of participants, the description of the types of analyses, and the definitions of each type of analysis. The reports involved studies of gabapentin used for medical reasons not approved for marketing by the US Food and Drug Administration, known as “off-label” uses.

**What Did the Researchers Do and Find?:**

The researchers identified trials sponsored by Pfizer relating to four off-label uses of gabapentin and examined the internal company protocols, statistical analysis plans, research reports, and the main publications related to each trial. The researchers then compared the numbers of participants randomized and analyzed for the main (primary) outcome and the type of analysis for efficacy and safety in both the internal research report and the trial publication. The researchers identified 21 trials, 11 of which were published RCTs that had the associated documents necessary for comparison.

The researchers found that in three out of ten trials there were differences in the internal research report and the main publication regarding the number of randomized participants. Furthermore, in six out of ten trials, the researchers were unable to compare the internal research report with the main publication for the number of participants analyzed for efficacy, because the research report either did not describe the primary outcome or did not describe the type of analysis. Overall, the researchers found that seven different types of efficacy analyses were described in the protocols, statistical analysis plans, and publications, including intention-to-treat analysis. However, the protocol or publication used six different descriptions for the intention-to-treat analysis, resulting in several important differences between the internal and published documents about the number of patients included in the analysis.

**What Do These Findings Mean?:**

These findings from a sample of industry-sponsored trials on the off-label use of gabapentin suggest that when compared to the internal research reports, the trial publications did not always accurately reflect what was actually done in the trial. Therefore, the trial publication could not be considered to be an accurate and transparent record of the numbers of participants randomized and analyzed for efficacy. These findings support the need for further revisions of the CONSORT statement, such as including explicit statements about the criteria used to define each type of analysis and the numbers of participants excluded from each type of analysis. Further guidance is also needed to ensure consistent terminology for types of analysis. Of course, these revisions will improve reporting only if authors and journals adhere to them. These findings also highlight the need for all individual patient data to be made accessible to readers of the published article.

**Additional Information:**

Please access these Web sites via the online version of this summary at http://dx.doi.org/10.1371/journal.pmed.1001378.

- For more information, see the CONSORT statement website
- The EQUATOR Network website is a resource center for the good reporting of health research studies and has more information about the SPIRIT initiative and the CONSORT statement

## Introduction

Intent to treat (ITT) analysis, which includes all participants in the groups to which they were randomized, is typically recommended as the primary type of analysis for randomized controlled trials (RCTs) [1]–[3]. An ITT analysis preserves the random assignment of participants to the study groups and thereby protects against selection bias while estimating the treatment effect, when no outcome data are missing. In addition to ITT, other types of analysis (e.g., per protocol analysis) are often conducted in RCTs. Such additional analyses, which include only a subset of all participants in the trial, can be used to assess sensitivity of findings compared to the primary analysis. Because each type of analysis estimates a different parameter (i.e., population effect) and exclusion of participants from an analysis can potentially bias its estimate (i.e., observed effect), details on the types of analysis and their definitions (i.e., criteria for including participants in the analysis), and the numbers of patients randomized and analyzed are necessary for interpreting the trial's findings [4].

Published reports of trials, and research, in general, must be an unbiased and accurate description of their conduct and findings [5]. Although the Consolidated Standards of Reporting Trials (CONSORT) statement has helped improve transparency in reporting methods and findings from RCTs [1],[3],[6], it has not been possible to identify the degree to which published reports accurately reflect the trial conduct. One of the areas that has not been well examined is how accurately trial publications describe the numbers of participants randomized and how they were analyzed. Existing research on consistency of criteria for including participants in the analysis and transparency in reporting focuses on two types of analysis, ITT and modified ITT (MITT) [7]–[13]; this research compares either the types of analysis across publications or what was planned in the trial protocol with what was reported in the publication [8]. We are unaware of research comparing what actually happened in the trial (i.e., patients included in internal study analyses) with what was reported in the trial publication.

Our objective was to compare, using a sample of industry-sponsored trials, the transparency and accuracy of reporting the numbers of participants, the description of types of analyses, and the definitions of each type of analysis (i.e., criteria for including participants in the analysis).

## Methods

### Data Sources

To achieve our study objective, we compared the description of the types of analysis and their definitions in the internal company protocol and research report (i.e., what was planned and what was done) to the corresponding publication (i.e., what was reported). The internal company documents used in our study were made available as part of litigation against Pfizer, Inc., (the defendants), by Kaiser Foundation Health Plan, Inc., (the plaintiffs), in Boston, Massachusetts [14]. We conducted our study as a scholarly pursuit independent of the litigation.

All trials included in our study were sponsored by Pfizer and Warner-Lambert's subsidiary Parke-Davis (Pfizer and Parke-Davis) and related to four off-label uses of gabapentin (i.e., indications not approved by the US Food and Drug Administration [FDA]) [15],[16]: migraine prophylaxis, and treatment of bipolar disorders, neuropathic pain, and nociceptive pain. We examined internal company documents and all trial publications that were made available to one of the authors (KD) for preparation of a report for the plaintiffs' lawyers as part of the litigation against Pfizer [15]. One additional trial publication was identified through an ad hoc bibliographic search [17]. Publications for trials included in our sample were dated between 1998 and 2008 in most cases [17]–[26], except for one trial publication in 1987 [27]. When there was more than one publication emanating from a trial, we specified one main publication for each trial on the basis of the following order of priority: full-length journal article; letter to the editor with study results; non-systematic review with pooled analysis; conference abstract.

For each trial, we examined internal company protocols, including amendments, and statistical analysis plans (SAPs), research reports, and the main publications. We used the label described in the internal company document to identify whether it was a protocol, SAP, or a research report. Internal company documents labeled “research report” were produced by or for Pfizer and Parke-Davis and included a detailed description of the methods, results of all statistical analyses in the trial, and a discussion. Research reports typically also included study protocols, protocol amendments, and SAPs in the appendices. We considered internal company documents labeled “Protocol” as the study protocol and those labeled “Inferential analysis plan,” “Statistical analysis plan,” or “Report and analysis plan” as the SAP.

We identified one main publication for 13 of 21 trials for the four indications [17]–[29]. We included in our analysis 11 published RCTs. We excluded one published trial because we did not have access to any internal company documents [28] and another published trial because it was not randomized and is thus, not applicable to comparisons we made for the types of analysis [29]. In addition, we excluded eight trials that were never published.

### Data Extraction and Comparisons

From the research report and the publication, we extracted data on participant flow (i.e., number of participants assessed and excluded for eligibility at screening, number randomized to treatment groups, number that received and did not receive the assigned treatment, number lost to follow-up, discontinuing intervention, and number included in the efficacy [per the publication-specified primary outcome] and safety analyses).

From the protocol, research report, and publication, we extracted data on the types of analysis described for the efficacy and safety outcomes, and the definition for each type of analysis (i.e., criteria for including participants in the analysis).

We made the following comparisons: (1) numbers of participants randomized and analyzed for efficacy (publication-specified primary outcome): research report versus publication (ten trials where both documents were available). (2) types of analyses for efficacy and safety, and their definitions: protocol versus research report (eight trials where both documents were available); protocol versus publication (nine trials where both documents were available); research report versus publication (ten trials where both documents were available).

One author (SSV) extracted data into an MS Access database and a second author (TL) verified all of the extracted data, by comparing it with the original documents. There was 97.5% agreement between the two authors (SV and TL) on data extracted from protocols and SAPs and 73% agreement on data extracted from research reports and publications. A third author (KD) independently verified, through reference to the original documents, all discrepancies between the first two abstractors and a 10% random sample of items (sample taken using a random number table) agreed upon by the first two abstractors (complete agreement for the 10% sample). We resolved disagreements among authors through discussion and consensus.

## Results

### Reporting on Numbers of Participants Randomized and Analyzed: Comparing Research Reports Versus Publications (Ten Trials Where Both Documents Were Available)

#### Number randomized

There was disagreement between the research report and the main publication on the number of participants randomized for three of ten trials (see Table 1). For one of the three trials, the research report described the completed study findings while the main publication was a conference abstract describing preliminary results [27]. We were unable to identify a reason for the disagreement in the remaining two trials [24],[26].

**Table 1:** Total number of participants randomized and number analyzed for efficacy per research report and publication for the publication-specified primary outcome.

Study ID	Number Randomized (Total)		Number Analyzed (Total) for Publication-Specified Primary Outcome		
	Research Report	Publication	Research Report	Publication	Type of Analysis
**Migraine prophylaxis**					
879-201 [27]	87	45^a^	Not reported^b^	33^a^	Not specified
945-220 [23]	145	145	Not reported^b^	87	MITT
**Bipolar disorders**					
945-209 [24]	118	117	114^b^^,^ c	114^c^	ITT
945-291 [26]	42	25	Not reported^b^	25	ITT
**Neuropathic pain**					
945-210 [18]	165	165	162	162	ITT
945-224 [19]	325	325	Not reported^d^	Not reported	Not specified
945-271 [17]	120	120	98	98	ITT
945-276 [20]	121	121	Not reported^b^	115	MITT
945-306 [25]	307	307	Not reported^d^	305	Evaluable
945-411 [21]	339	339	339	339	ITT
No ID Gorson [22]	No research report^e^	40^a^	Not available^e^	Not reported^a^	Not specified

a The only published report available to us for study 879-201, which we used as its “main publication,” was a conference abstract describing preliminary results of analyses from the trial [27]. The only published report available to us for the study, No ID Gorson, which we used as its “main publication,” was a letter to the editor describing results from the trial [22]. We did not use a conference abstract, a report describing preliminary results of analyses, or a letter to the editor as the “main publication” for any other trial listed in this table.

b We could not compare the internal company research report and publication because the internal company research report did not report on the publication-specified primary outcome.

c The numbers presented are for Young Mania Rating Scale, which was one of two outcomes considered “primary.” The corresponding numbers for the other outcome considered “primary,” the Hamilton Depression Rating Scale (HAM-D) are as follows: ITT: 113 in the research report and 113 in publication.

d We could not compare the internal company document and publication because the internal company research report did not specify the type of analysis specified in the publication, even though the outcomes were the same.

e A draft manuscript sent to Pfizer and Parke-Davis by the author reports a total of 53 patients were allocated in the trial and 40 participants were analyzed for efficacy.

#### Number analyzed

For six of ten trials, we were unable to compare the research report with the main publication for the number of participants analyzed for efficacy, because the research report either did not describe the publication-specified primary outcome or did not describe the publication-specified type of analysis (see Table 1 footnotes). There was agreement for four of ten trials.

Because of substantial variations in terminology within documents related to the same trial as well as across trials, we decided not to make comparisons between the research report and the main publication on numbers related to participant flow. We made this decision after two authors (SV and TL) independently attempted to reconcile variations in terminology followed by a consensus discussion among all authors.

Two examples of variation in terminology within documents from the same trial follow. For study 945-220, the research report listed 24 patients in the gabapentin group and nine in the placebo group under the heading “Premature Discontinuation”; and the main publication specified that 24 patients in the gabapentin group and nine in the placebo group were “Withdrawn” and provided “Reasons for Discontinuation.” [23]. For study 945-276, the research report specified that 21 patients in the gabapentin group and ten in the placebo group “dropped out” but did not provide a definition for “drop-outs”; the main publication described that 21 patients in the gabapentin group and nine patients in the placebo group “Discontinued Intervention.” [20]

Variation in terminology may affect study findings, for example in study 945-220, MITT was described in the publication as follows: “This population included any patient who was randomized, took at least one dose of study medication during SP2 [Stabilization Period 2], maintained a stable dose of 2,400 mg/day during SP2, had baseline migraine headache data and at least 1 day of migraine headache evaluations during SP2” [23].

If “Premature discontinuation” had meant that participants discontinued the assigned treatment but outcomes were still assessed during “stabilization period 2” of follow-up then they would be eligible for inclusion in the MITT analysis described in the publication.

In contrast, if “Premature discontinuation” had meant that the participants were no longer followed up for assessing outcomes then they would not be eligible for inclusion in the MITT analysis (in all cases where the “premature discontinuation” resulted in no outcomes being assessed during stabilization period 2).

Thus, the two interpretations would mean that different subsets of participants are included in the analysis, even while using the same definition for the type of analysis, thereby leading to different findings.

### Reporting on the Types of Analysis for Efficacy and Safety: Comparing Protocols Versus Research Reports (Eight Trials Where Both Documents Were Available)

We observed extensive variability and inconsistency (i.e., disagreements) among documents (i.e., protocol, research report, SAPs, and publication) within the same trial and across trials for the types of analyses that were specified as well as their definitions. An ITT analysis was specified in the protocol for five of eight trials and in the research report for seven of eight trials. Both documents described a definition for ITT in three of eight trials and there was a disagreement in the definition for two of three trials (see Table 2). An example of a disagreement in the definition is shown below.

**Table 2:** Agreement among documents from the same trial on definitions of ITT analysis and safety analysis.

Documents Compared and Criteria Assessed in Each Comparison	*n* Published Trials
**Protocol versus research report**	**8 total**
*ITT analysis*	
Specified in protocol	5/8
Specified in research report	7/8
Described definition in both protocol and research report	3/8
Agreement on definition of ITT between protocol and research report	1/3
Disagreement on definition of ITT between protocol and research report	2/3
*Safety analysis*	
Specified in protocol	6/8
Specified in research report	7/8
Described definition in both protocol and research report	5/8
Agreement on definition of safety analysis between protocol and research report	3/5
Disagreement on definition of safety analysis between protocol and research report	2/5
**Protocol versus publication**	**9 total**
*Definition for ITT analysis*	
Specified in protocol	5/9
Specified in publication	4/9
Described definition in both protocol and publication	3/9
Agreement on definition of ITT between protocol and publication	1/3
Disagreement on definition of ITT between protocol and publication	2/3
*Definition for safety analysis*	
Specified in protocol	6/9
Specified in publication	3/9
Described definition in both protocol and publication	2/9
Agreement on definition of safety analysis between protocol and publication	1/2
Disagreement on definition of safety analysis between protocol and publication	1/2
**Research report versus publication**	**10 total**
*Definition for ITT analysis*	
Specified in research report	9/10
Specified in publication	6/10
Described definition in both research report and publication	4/10
Agreement on definition of ITT between research report and publication	2/4
Disagreement on definition of ITT between research report and publication	2/4
*Definition for safety analysis*	
Specified in research report	9/10
Specified in publication	4/10
Described definition in both research report and publication	4/10
Agreement on definition of safety analysis between research report and publication	3/4
Disagreement on definition of safety analysis between research report and publication	1/4

#### Study 945-224

The protocol defined an ITT analysis as follows: “…this includes all patients randomized to treatment who received at least 1 dose of study medication.”

The methods section in the research report for the same trial defined ITT as follows: “The ITT population was defined as all patients randomized who received at least 1 dose of study medication in the double-blind phase.”

The results section in the research report defined ITT as follows: “This population comprised all patients who received at least 1 dose of study medication and who had an observation for the primary efficacy parameter at baseline.”

We also found disagreements in the definition for safety analysis between the protocol and research report for the same eight trials. The definition for a safety analysis was described in both documents in the case of five of eight trials; there was disagreement on the definition for two of five of these trials (see Table 2 and Text S1).

### Reporting on the Types of Analysis for Efficacy and Safety: Comparing Protocols Versus Publications (Nine Trials Where Both Documents Were Available)

Seven different types of analyses for efficacy were described in protocols (including amendments and SAPs) and publications across the included trials (see Table 3). The definitions for the types of analysis were not always consistent across trials or between the protocol and publication for the same trial.

**Table 3:** Summary of criteria used for including participants in seven types of analyses for efficacy as described in protocols, statistical analysis plans, and publications across the nine included trials.

Criteria to Define Types of Analysis	Type of Analysis for Efficacy^a^						
	“ITT”^b^	“MITT”^b^	“Per Protocol”^c^	“Evaluable”^b^	“Evaluable for Efficacy”^c^	“Efficacy Evaluable”^d^	“Population to be Analysed”^c^
All patients randomized to treatment	X	X	—	X	—	—	X
Completed treatment at minimum dose and/or for a minimum duration	X	X	—	X	X	X	X
Data available for outcome variable at baseline or screening	X	X	—	X	X	X	—
Data available after randomization for a specified or unspecified number of visits/days	X	X	—	X	X	X	X
Completed minimum duration of follow-up after randomization and/or in the baseline period	X	—	—	—	—	X	—
No protocol violations or variations	—	—	X	X	—	—	—
Included in the ITT analysis	—	—	X	X	—	—	—
Meet criteria for MITT analysis	—	—	—	—	X	X	—
Meet criteria for treatment compliance, concomitant medications, or both	—	—	—	—	X	X	—

a This table summarizes data presented in Tables 4 and 5 and Text S1's table 1. Along the top row in this table, we show every type of efficacy analysis described in the protocols, SAPs, and publications across all nine trials for which we compared these documents. The first column on the left lists the criteria used to define the types of analysis across all studies. For each type of analysis, an “X” denotes that the criterion was applied in at least one of the documents for any of the nine trials we examined. For example, the second column summarizes the five criteria used across all documents and trials to define ITT: in Table 4, four criteria were used in different combinations to define ITT analysis; in Text S1's table 1, one additional criterion was used in the SAP.

b This type of analysis was specified protocols, SAPs, and publications for the trials we examined (Tables 4 and 5 and Text S1's table 1).

c This type of analysis was specified only in the protocol and publications for some of the trials we examined (see Table 5).

d This type of analysis specified only in SAPs for some of the trials we examined (see Text S1's table 1).

CGIS, clinical global impression of severity; HAM-D, Hamilton Depression Rating Scale; YMRS, Young Mania Rating Scale.

Six different definitions for ITT were described in the protocols, SAPs, and publications we examined (see tables 4 and 1 in Text S1). In neither type of document did the definition for ITT ever match its widely accepted description (i.e., all randomized patients in the groups they were assigned) [1]–[3]. For three of nine trials, a definition was available in both the protocol and publication; there was disagreement for two of the three trials. An example of a disagreement in the definition is shown below.

**Table 4:** Criteria used to define ITT analysis in protocols (Pro) and publications (Pub).

Criteria Used to Define ITT Analysis	Trials Reporting an ITT Analysis																	
	879-201		945-220		945-209		945-210		945-224		945-271		945-306		945-411		No ID- Gorson	
	Pro	Pub [27]	Pro	Pub [23]	Pro	Pub [24]	Pro	Pub [18]	Pro	Pub [19]	Pro	Pub [17]	Pro	Pub [25]	Pro	Pub [21]	Pro	Pub [22]
Did not specify this type of analysis	**X**	**X**	**X**	**X**	—	—	—	—	—	**X**	—	—	**X**	**X**	—	—	**X**	**X**
Type of analysis specified but no details on criteria were reported	—	—	—	—	—	—	—	—	—	—	**X**	—	—	—	—	—	—	—
All patients randomized to treatment	—	—	—	—	**X**	**X**	**X**	**X**	**X**	—	—	**X**	—	—	—	**X**	—	—
Completed treatment at minimum dose and/or for a minimum duration	—	—	—	—	—	**X**	**X**	**X**	**X**	—	—	**X**	—	—	**X**	**X**	—	—
Data available for outcome variable at baseline or screening	—	—	—	—	—	—	—	—	—	—	—	—	—	—	—	**X**	—	—
Completed minimum duration of follow-up after randomization and/or in baseline period	—	—	—	—	**X**	—	—	—	—	—	—	—	—	—	—	—	—	—

To be eligible for inclusion in the ITT analysis, participants had to meet the criteria that are marked with an “X”. For example, per the description of ITT in the publication for study 945-209 [24], data from participants who were randomized into the trial and met criteria related to completing treatment at a minimum dose and/or for a minimum duration were included in the ITT analysis (“The efficacy analyses were carried out on the intent-to-treat (ITT) population that included all randomized patients who received at least one dose of study medication.” [24]).

#### Study 945-209

The protocol defined ITT as follows: “A secondary population will be the Intent-to-Treat population which is defined as all patients randomized to treatment and who have at least 1 postrandomization visit.”

The publication defined ITT as follows: “The efficacy analyses were carried out on the intent-to-treat (ITT) population that included all randomized patients who received at least one dose of study medication.” [24]

There were disagreements between the protocol and publication for other types of analysis for efficacy, including “modified intent to treat (MITT),” “per protocol,” “evaluable,” “evaluable for efficacy” and “Population to be analysed” (see Table 5).

**Table 5:** Criteria used to define additional types of analysis in protocols (Pro) and publications (Pub).

Criteria Used to Define Type of Analysis	Trials Reporting Additional Types of Analysis^a^															
	“MITT”		“Per Protocol”		“Evaluable”								“Evaluable for Efficacy”		“Population to Be Analysed”	
	945-220^b^		945-271		945-209		945-210		945-306		945-411		945-220		945-306	
	Pro	Pub [23]	Pro	Pub [17]	Pro	Pub [24]	Pro	Pub [18]	Pro	Pub [25]	Pro	Pub [21]	Pro	Pub [23]	Pro	Pub [25]
Did not specify this type of analysis	—	—	—	—	—	**X**	—	**X**	**X**	—	**X**	—	—	**X**	—	**X**
Type of analysis specified but no details on criteria were reported	—	—	**X**	—	—	—	—	—	—	—	—	—	—	—	—	—
All patients randomized to treatment	**X**	**X**	—	—	**X**	—	—	—	—	**X**	—	—	—	—	**X**	—
Completed treatment at minimum dose and/or for a minimum duration	**X**	**X**	—	—	**X**	—	**X**	—	—	**X**	—	**X**	**X**	—	**X**	—
Data available for outcome variable at baseline or screening	**X**	**X**	—	—	**X**	—	**X**	—	—	**X**	—	**X**	**X**	—	—	—
Data available after randomization for a specified or unspecified number of visits/days	**X**	**X**	—	—	**X**	—	**X**	—	—	**X**	—	**X**	**X**	—	**X**	—
No protocol violations or variations	—	—	—	**X**	**X**	—	—	—	—	—	—	**X**	—	—	—	—
Included in the intent to treat analysis	—	—	—	**X**	—	—	—	—	—	—	—	**X**	—	—	—	—
Meet criteria for MITT analysis	—	—	—	—	—	—	—	—	—	—	—	—	**X**	—	—	—
Meet criteria for treatment compliance, concomitant medications, or both	—	—	—	—	—	—	—	—	—	—	—	—	**X**	—	—	—

a The table does not include an additional type of analysis (“Efficacy evaluable”) specified in the SAP for study 945-220. See Text S1 for details.

b To be eligible for inclusion in a type of analysis, participants had to meet the criteria that are checked. For example, per the description of modified ITT in the publication for study 945-220 [23], data from participants who were randomized into the trial and met criteria related to completing treatment at a minimum dose and/or for a minimum duration, and availability of measurements for the outcome variable at baseline and during follow-up, were included in the modified ITT analysis (“This population included any patient who was randomized, took at least one dose of study medication during SP2 [Stabilization Period 2], maintained a stable dose of 2400 mg/day during SP2, had baseline migraine headache data and at least 1 day of migraine headache evaluations during SP2.” [23]).

Criteria for including participants in analyses of safety outcomes were variably described in the protocols and publications across trials. Five different definitions for the safety analysis were used in protocols, SAPs, and publications across all included trials (see Tables 2 and 6). For two of nine trials, the definition for safety analysis was described in both documents and there was disagreement on the definition in one of the two trials.

**Table 6:** Comparison of protocol (Pro), SAPs, and publications (Pub) for description of criteria used to determine inclusion of trial participants in safety analysis.

Criteria Used to Define Safety Analysis	Safety Analysis^a^^,^ b																						
	879-201			945-220			945-209		945-210			945-224			945-271		945-306			945-411		No ID - Gorson	
	Pro	SAP	Pub [27]	Pro	SAP	Pub [23]	Pro	Pub [24]	Pro	SAP	Pub [18]	Pro	SAP	Pub [19]	Pro	Pub [17]	Pro	SAP	Pub [25]	Pro	Pub [21]	Pro	Pub [22]
Did not explicitly specify or describe criteria for safety analysis	**X**	**X**	**X**	**—**	**—**	**X**	**—**	**X**	**—**	**X**		**—**	**—**	**X**	**X**	**—**	**—**	**—**	**X**	**—**	**—**	**X**	**X**
All patients randomized	**—**	**—**	**—**	**—**	**—**	**—**	**—**	**—**	**—**	**—**	**X**	**—**	**X**	**—**	**—**	**X**	**—**	**X**	**—**	**—**	**—**	**—**	**—**
Completed treatment at minimum dose and/or for a minimum duration	**—**	**—**	**—**	**X**	**X**	**—**	**X**	**—**	**X** c	**—**	**—**	**X** c	**X**	**—**	**—**	**X**	**X**	**X**	**—**	**X** c	**X**	**—**	**—**
Data available after randomization for a specified or unspecified number of visits/days	**—**	**—**	**—**	**X** d	**X**	**—**	**—**	**—**	**—**	**—**	**—**	**—**	**—**	**—**	**—**	**—**	**—**	**—**	**—**	**—**	**—**	**—**	**—**

a Neither the protocol, SAP, nor the publication for Study 879-201 described criteria used to determine inclusion of trial participants in safety analysis.

b Neither the protocol nor the publication for Study No ID - Gorson described criteria used to determine inclusion of trial participants in safety analysis.

c “All patients who have taken study medication will be included in the evaluation of safety data.”

d “Any patient who has taken at least one dose of study medication and has had an opportunity to report an adverse experience via clinic visit, telephone contact, etc. will be evaluable for the safety analysis.”

### Reporting on the Types of Analysis for Efficacy and Safety: Comparing Research Reports Versus Publications (Ten Trials Where Both Documents Were Available)

Agreement between the research report and publication on description of types of analysis for efficacy varied across the trials included for this comparison. For example, while a definition for ITT was available in both the research report and publication in four trials there was disagreement for two of the four trials. There was also frequent disagreement between the research report and publication for other types of analyses for efficacy (data shown in Text S1).

The research reports described criteria for including participants in analyses of safety more often than publications, and in greater detail. For four of the ten trials, a definition for safety analysis was described in both documents; there was disagreement for one of the four trials.

### Nature of Disagreements across the Trial Documents

The most commonly observed form of disagreement on types of analyses across the documents was omission from the publication of one or more types of analysis for efficacy specified in the trial protocol (five of nine trials with both documents) or research report (seven of ten trials with both documents).

## Discussion

In our sample of industry-supported trials in off-label uses of gabapentin, we observed discrepancies between what was reported in trial publications and what was described in internal company research reports. In this regard, we found that the trial publication was not a transparent, or accurate (presuming that the research report truly describes the facts), record for the numbers of participants randomized and analyzed for efficacy. In three of ten trials in our sample, the number of participants randomized in the trial, as specified in the “main publication” [24],[26],[27], was not the same as that described in the research report. The “main publication” was a full-length journal article for two of the three trials with a disagreement in the number of participants randomized in the trial [24],[26], and a conference abstract describing preliminary results for the third trial [27]. In one case, the description in the publication did not include data from 40% of participants actually randomized in the trial (as described in the research report; see Table 1) [26]. There was such wide variation in describing the participant flow in the included trials, even among documents for the same trial, that we were unable to summarize what we found.

In addition, we observed extensive variability, both among documents for the same trial and across trials, in the types of analyses specified for efficacy and safety as well as in the criteria for including participants in each type of analysis; this is consistent with Chan's findings comparing protocols and publications [8]. Our study extends comparisons of the protocol and publication to comparisons of analyses presented in internal company research reports and analyses presented publicly.

We are concerned that, even for commonly used types of analysis such as ITT, a number of different definitions were used across trials included in our sample. Trial findings may be sensitive to the alternative definitions used for the type of analysis, i.e., analyses using different definitions of the type of analysis can include different subsets of participants, thereby leading to different findings.

Because internal company documents are not generally available, our study provides a unique snapshot of how well a publication may reflect what is known by the company. Since doctors rely on publications for information about a drug's effectiveness for off-label indications, our study raises particularly important questions that may be applicable more broadly to other drugs like gabapentin, which has been prescribed so frequently for off-label indications [30].

Because we included a small number of trials for off-label indications sponsored by a single company using documents released as part of litigation, our findings may not apply to other industry-sponsored trials, to trials for other off-label indications, or to trials sponsored by other entities. We did not undertake a systematic literature search to identify any additional publications that may have described results from the included trials. In nearly every case, since the documents associated with each trial were obtained as part of the legal discovery process, we believe that we have a complete list of publications for each trial.

We did not have access to the protocol for two trials and the research report for one trial, and were unable to compare them with the corresponding trial publication. For one trial (study 879–201), we used a conference abstract describing preliminary results [27] as the “main publication” for our comparisons with the protocol and research report. Because the only information the public has about the findings from study 879-201 is from the conference abstract [27], we believe that our observations using it are relevant in the context of our study. We are not able to determine the cause for missing data and inadequate reporting of data for any of the trial documents we examined. For example, editing to meet space constraints may have been responsible for omission of information from published reports.

In addition, we did not assess whether disagreements in descriptions of types of analyses resulted in different participants being analyzed for efficacy and safety. We also did not examine the impact of the observed disagreements on the effectiveness of gabapentin for the indications specified in our study, as meaningful assessment would have required a systematic review of each trial topic. We encourage this important area of research, however.

The discrepancies in description of types of analyses we observed in our study are not unique to trials sponsored by for-profit entities such as pharmaceutical companies. Using trial protocols obtained from institutional review boards and corresponding publications, previous research has shown that disagreements such as those we described in our study can be observed in trials with funding from both for-profit and not-for-profit entities [8].

Our findings support recommendations that simply stating in the publication that an analysis used ITT is not adequate [2],[3]. Among the trials that reported a planned ITT analysis (about half of the trials in our analysis) in the protocol, the definition of ITT (i.e., which participants were included in the ITT analysis) varied. In no case did the documents describe an ITT analysis according to the widely accepted definition, i.e., all randomized participants in the groups to which they were assigned. Because ITT is recommended for the primary analysis of randomized trials [1]–[3], modifying its definition, whether across studies or within a study, defeats the purpose of a standard.

Even if the definition of an ITT is “all randomized participants analyzed as part of the group to which they were assigned,” results from a single trial can still vary depending on the method used to impute or account for missing data (e.g., patients who missed a visit). Indeed, a recent study showed that even among reports of methodological studies, the definition of ITT varied with regard to how missing outcome data are handled in the analyses [31]. A proposed revision to the CONSORT statement reportedly will include a requirement for describing any imputation of missing data used to conduct an ITT analysis [32].

On the basis of our findings, we suggest that the following items be considered for inclusion as recommended standards for reporting trials in subsequent revisions to the CONSORT statement: (1) explicitly state all protocol-specified type(s) of analysis, or the analysis populations, for analyses of primary, secondary, and safety outcomes; (2) clearly state the criteria used to define each type of analysis, if more than one is specified; (3) explicitly specify the numbers of participants who do not meet each of the stated criteria, and are therefore excluded, for each type of analysis; (4) specify terminology for consistent use across trials, for example, for types of analysis, participants who do not receive treatment as assigned (“treatment dropouts”), and patients not included in the analysis (“analysis dropouts”) [4]; (5) recommend that the individual patient data used for all analyses described in the trial report be made accessible to the biomedical community along with the published article.

In addition to being relevant to revisions of the CONSORT statement, the recommendations listed above are also relevant to the Standard Protocol Items for Randomized Trials (SPIRIT) Initiative, which includes items on methods to handle protocol deviations and missing data but does not explicitly require a detailed definition for the type of analysis for efficacy and safety [33].

Although revisions to reporting standards can contribute to improving transparency of reporting, they cannot assure accuracy of reporting as noted above. A variety of initiatives could improve the situation, although there is no way to completely eliminate deliberate fraud. Transparency, through public availability of trial protocols, research reports, and results, is a possible solution [34]. But existing initiatives to this end do not go far enough to ensure transparency.

Our work emphasizes that research reports, which are similar to “clinical study reports” described in another context [35], provide a more comprehensive and presumably, a more accurate narrative of the conduct of a trial and its findings than a journal publication. For example, the research reports for trials in our sample more often provided details about the definitions for types of analyses, while the corresponding trial publications omitted such details. The research reports are typically considered confidential documents and are rarely available to the public or the wider biomedical community. Our findings lend support to the principle that such “research reports” or “clinical study reports” must be readily accessible to the biomedical community and the public [35] so that the veracity of what is reported in the publications can be assessed.

Current US legal requirements for reporting study findings are inadequate both in scope and detail. FDA approval is not required for off-label use of drugs. Although information on trials in off-label uses of drugs may be submitted to the FDA, the agency's reviews of the trial data are made publicly available only if the application to market the drug is approved [36]–[38]. Consequently, for trials of off-label uses of drugs that are not submitted to the FDA, or that are submitted to the FDA as part of a withdrawn or unsuccessful application to market the drug, the only publicly available source of information about effectiveness accessible to most decision-makers is the published literature.

The FDA Amendments Act of 2007 requires that summary results for trials of off-label uses of approved drugs, even some investigational new drug-exempt trials, must be posted on ClinicalTrials.gov, for all trials not completed before 2007 [39],[40]. Having results of trials in off-label uses available publicly allows comparison of the sponsor's trial data with information in publications [41]–[43]. It is not clear to us, however, that having summary data available through ClinicalTrials.gov is sufficient to allow confirmation of the trial findings by other investigators. Certainly, the final dataset along with the full clinical study report from industry-sponsored trials for approved drugs should be made available by the FDA to the public for this purpose [44]. The European Medicines Agency (EMA), in contrast, makes publicly accessible its summary (i.e., European Public Assessment Report) and certain documents submitted to the agency as part of applications requesting authorization to market drugs [35],[45]–[47]. Other investigators have successfully obtained documents from the EMA, including clinical study reports, to verify claims in publications about a drug's effectiveness [35].

Our findings highlight the need for standardizing the definitions for various types of analyses that may be conducted to assess intervention effects in clinical trials, delineating the circumstances under which different types of analyses are meaningful, and educating those who are involved in conducting and reporting trials such that the standards are consistently adopted. We believe that our findings lend support to policy considerations such as extending mandatory registration to include all clinical trials [48],[49], making full trial protocols and trial data publicly available through trial registration or other means, and ensuring that regulations pertaining to compulsory reporting of results apply both to trials conducted for regulatory authority-approval and to trials in off-label indications of interventions. Health interventions are administered to members of the public on the basis of trial findings. It is time for the balance of power in access to information from clinical trials to be shifted from those sponsoring the trials to the public at large.

## Supporting Information

Text S1 **Additional data tables.** Differences in reporting of analyses in internal company documents versus published trial reports: comparisons in industry-sponsored trials in off-label uses of gabapentin.
(DOC)

## Abbreviations

CONSORT: Consolidated Standards of Reporting Trials
FDA: US Food and Drug Administration
ITT: intention to treat
MITT: modified intention to treat
RCT: randomized controlled trial
SAP: statistical analysis plan
