# Supplementary material for: Differences in Reporting of Analyses in Internal Company Documents Versus Published Trial Reports: Comparisons in Industry-Sponsored Trials in Off-Label Uses of Gabapentin
Source: PLoS Med. 2013 Jan 29;10(1):e1001378. doi: 10.1371/journal.pmed.1001378 (PMC3558476; doi:10.1371/journal.pmed.1001378)
Supplement: Text S1 — Additional data tables. Differences in reporting of analyses in internal company documents versus published trial reports: comparisons in industry-sponsored trials in off-label uses of gabapentin. (DOC) [file pmed.1001378.s001.doc]

**Text S1.** Additional data tables for Vedula SS, Li T, Dickersin K. Differences in reporting of analyses in internal company documents versus published trial reports: comparisons in industry-sponsored trials in off-label uses of gabapentin. PLOS Med 10(1): e1001378. doi: 10.1371/journal.pmed.1001378.

**Contents**

| Table 1 | Types of analyses and criteria used to define each type of analysis in statistical analysis plans (SAPs) and publications | Page 2 |
| --- | --- | --- |
| Table2a | Types of analyses and criteria used to define each type of analysis in internal company research reports and publications (intent to treat and modified intent to treat) | Page 3 |
| Table2b | Types of analyses and criteria used to define each type of analysis in internal company research reports and publications (evaluable, efficacy evaluable, week one completers, and per protocol) | Page 5 |
| Table 3 | Comparison of internal company research reports and publications for description of criteria used to determine inclusion of trial participants in safety analysis | Page 7 |
| Table 4 | Verbatim text from documents we examined for trials specified in Table 2 of the article as having had a disagreement between protocol and research report on description of definition for intent to treat analysis | Page 9 |
| Table 5 | Verbatim text from documents we examined for trials specified in Table 2 of the article as having had a disagreement between protocol and research report on description of definition for safety analysis | Page 10 |
| Table 6 | Verbatim text from documents we examined for trials specified in Table 2 of the article as having had a disagreement between protocol and publication on description of definition for intent to treat analysis | Page 11 |
| Table 7 | Verbatim text from documents we examined for trials specified in Table 2 of the article as having had a disagreement between protocol and publication on description of definition for safety analysis | Page 12 |
| Table 8 | Verbatim text from documents we examined for trials specified in Table 2 of the article as having had a disagreement between research report and publication on description of definition for intent to treat analysis | Page 13 |
| Table 9 | Verbatim text from documents we examined for trials specified in Table 2 of the article as having had a disagreement between research report and publication on description of definition for safety analysis | Page 14 |
| References |  | Page 15 |

**Table 1. Types of analyses and criteria used to define each type of analysis in statistical analysis plans (SAPs) and publications**

| **Criteria to define types of analysis** | **“Intent to Treat”** | | | | | | **“Modified Intent to Treat”** | | **“Evaluable”** | | | | **“Efficacy evaluable”** | |
| --- | --- | --- | --- | --- | --- | --- | --- | --- | --- | --- | --- | --- | --- | --- |
|  | **945-210** | | **945-224** | | **945-306** | | **945-220** | | **945-210** | | **945-306** | | **945-220** | |
|  | SAP | Publication^1^ | SAP | Publication^2^ | SAP | Publication^3^ | SAP | Publication^4^ | SAP | Publication^1^ | SAP | Publication^3^ | SAP | Publication^4^ |
| Did not specify this type of analysis |  |  |  | **X** |  | **X** |  |  |  | **X** | **X** |  |  | **X** |
| All patients randomized to treatment |  | **X** | **X** |  | **X** |  | **X** | **X** |  |  |  | **X** |  |  |
| Completed treatment at minimum dose and/or for a minimum duration | **X** | **X** | **X** |  | **X** |  | **X** | **X** | **X** |  |  | **X** | **X** |  |
| Data available for outcome variable at baseline or screening | **X** |  | **X** |  |  |  | **X** | **X** | **X** |  |  | **X** | **X** |  |
| Data available after randomization for a specified or unspecified number of visits/days | **X** |  | **X** |  |  |  | **X** | **X** | **X** |  |  | **X** | **X** |  |
| Completed minimum duration of follow-up after randomization and/or in baseline period |  |  |  |  |  |  |  |  |  |  |  |  | **X** |  |
| Did not use concomitant medication for the study condition |  |  |  |  |  |  |  |  |  |  |  |  | **X** |  |
| Meet criteria for modified intent to treat (MITT) analysis |  |  |  |  |  |  |  |  |  |  |  |  | **X** |  |

**Table 2a. Types of analyses and criteria used to define each type of analysis in internal company research reports and publications** (“intent to treat”)

| **Criteria to define types of analysis** | **“Intent to Treat”** | | | | | | | | | | | | | | | | | | | |
| --- | --- | --- | --- | --- | --- | --- | --- | --- | --- | --- | --- | --- | --- | --- | --- | --- | --- | --- | --- | --- |
|  | **879-201** | | **945-220** | | **945-209** | | **945-291** | | **945-210** | | **945-224** | | **945-271** | | **945-276** | | **945-306** | | **945-411** | |
|  | Research report | Publication^10^ | Research report | Publication^4^ | Research report | Publication^5^ | Research report | Publication^6^ | Research report | Publication^1^ | Research report | Publication^2^ | Research report | Publication^7^ | Research report | Publication^8^ | Research report | Publication^3^ | Research report | Publication^9^ |
| Did not specify this type of analysis |  | **X** | **X** | **X** |  |  |  |  |  |  |  | **X** |  |  |  |  |  | **X** |  |  |
| Type of analysis specified but no details on criteria were reported |  |  |  |  | **X^b^** |  |  | **X** |  |  |  |  |  |  |  |  |  |  |  |  |
| All patients randomized to treatment |  |  |  |  |  | **X** | **X** |  | **X** | **X** |  |  | **X** | **X** |  |  | **X** |  |  | **X** |
| Completed treatment at minimum dose and/or for a minimum duration | **X^a^** |  |  |  |  | **X** | **X** |  | **X** | **X** | **X^c^** |  | **X** | **X** | **X** | **X** | **X** |  | **X** | **X** |
| Data available for outcome variable at baseline or screening |  |  |  |  |  |  |  |  |  |  | **X^c^** |  |  |  |  |  |  |  | **X** | **X** |
| Data available after randomization for a specified or unspecified number of visits/days |  |  |  |  |  |  | **X** |  |  |  |  |  |  |  | **X** |  |  |  |  |  |
| Completed minimum duration of follow-up after randomization and/or in baseline period |  |  |  |  |  |  |  |  |  |  |  |  |  |  |  |  |  |  |  |  |
| Data available for an unspecified time-point (i.e., baseline/follow-up) | **X^a^** |  |  |  |  |  |  |  |  |  |  |  |  |  |  |  |  |  |  |  |
| Not randomized; treated on an open label basis (“Breaking code”) | **X^a^** |  |  |  |  |  |  |  |  |  |  |  |  |  |  |  |  |  |  |  |

**Footnotes for Table 2a**

a The research report included inconsistent descriptions for the type of analysis in the Methods and Results sections. We used data in the Results section, specifically Table 7 and text on Page 18.

Methods section

Page 11 “All patients were included in the analyses, even though one patient in each treatment group did not receive study medication.”

“Patients were excluded from efficacy if they continued taking prophylactic migraine medication or did not stop it at least one month before start of treatment.”

Page 12 “An intent-to-treat analysis included all randomized and treated patients was performed, using the response ratio as the analysis variable and the treatment as a grouping factor (t-test).”

Results section

Page 18 “Two patients in each group were excluded from the intent-to-treat analysis because no data on the number of attacks are available or because they had never taken the test medication which had been handed out to them.” Table 7 indicates that 2 people in each group were excluded because “No data available” and 2 more excluded in gabapentin group because of “Breaking code”. Thus, a total of 6 participants were excluded.

“The two patients treated with gabapentin on an open label basis were excluded from all efficacy analysis.”

b A letter to the investigators dated July 28, 1998, stated that the “intent-to-treat population consisted of 117 patients randomized, of whom 114 had a post-randomization observation on either placebo (n = 59) or gabapentin (n = 55).

c The research report included inconsistent descriptions for the type of analysis in the Synopsis, Methods and Results sections. We used data from Page 44 of the research report (Results section).

Synopsis

Page 8 “all patients randomized who received at least 1 dose of study medication in the double-blind phase and who had at least an observation for the primary efficacy parameter at baseline.”

Methods

Page 31 “The ITT population was defined as all patients randomized who received at least 1 dose of study medication in the double-blind phase. Patients who had neither observations for the primary efficacy parameter at baseline nor during the study were to be excluded from the ITT population.”

Results

Page 45 “The primary patient sample was the ITT population. This population comprised all patients who received at least 1 dose of study medication and who had an observation for the primary efficacy parameter at baseline or during the double-blind treatment period.”

**Table 2b. Types of analyses and criteria used to define each type of analysis in internal company research reports and publications** (“modified intent to treat,” “per protocol,” “evaluable,” “efficacy evaluable,” and “week one completers”)

| **Criteria to define types of analysis** | **“Modified intent to treat”**^a^ | | | | **“Per protocol”** | | | | **“Evaluable”** | | | | | | | | | | **“Efficacy evaluable”** | | **“Week one compl-eters”** | |
| --- | --- | --- | --- | --- | --- | --- | --- | --- | --- | --- | --- | --- | --- | --- | --- | --- | --- | --- | --- | --- | --- | --- |
|  | **945-220** | | **945-276** | | **945-291** | | **945-271** | | **879-201** | | **945-209** | | **945-210** | | **945-306** | | **945-411** | | **945-220** | | **945-209** | |
|  | Research report | Publication^4^ | Research report | Publication^8^ | Research report | Publication^6^ | Research report | Publication^7^ | Research report^b^ | Publication^10^ | Research report | Publication^5^ | Research report | Publication^1^ | Research report | Publication^3^ | Research report | Publication^9^ | Research report | Publication^4^ | Research report | Publication^5^ |
| Did not specify this type of analysis |  |  | **X** |  |  | **X** |  |  |  | **X** | **X** | **X** |  | **X** | **X** |  |  |  |  | **X** |  | **X** |
| Type of analysis specified but no details on criteria were reported |  |  |  |  |  |  |  |  |  |  |  |  |  |  |  |  |  |  |  |  |  |  |
| All patients randomized to treatment | **X** | **X** |  |  |  |  |  |  |  |  |  |  |  |  |  | **X** |  |  |  |  |  |  |
| Completed treatment at minimum dose and/or for a minimum duration | **X** | **X** |  | **X** |  |  |  |  | **X** |  |  |  | **X** |  |  | **X** |  | **X** | **X** |  | **X** |  |
| Data available for outcome variable at baseline or screening | **X** | **X** |  |  |  |  |  |  |  |  |  |  | **X** |  |  | **X** |  | **X** | **X** |  | **X** |  |
| Data available after randomization for a specified or unspecified number of visits/days | **X** | **X** |  | **X** |  |  |  |  |  |  |  |  | **X** |  |  | **X** |  | **X** | **X** |  | **X** |  |
| Completed minimum duration of follow-up after randomization and/or in baseline period |  |  |  |  |  |  |  |  |  |  |  |  |  |  |  |  |  |  | **X** |  |  |  |
| Did not use concomitant medication for the study condition, meet criteria for compliance, or both |  |  |  |  | **X** |  |  |  | **X** |  |  |  |  |  |  |  | **X** | **X** | **X** |  |  |  |
| Meet criteria for modified intent to treat (MITT) analysis |  |  |  |  |  |  |  |  |  |  |  |  |  |  |  |  |  |  | **X** |  |  |  |
| Meet criteria for intent to treat analysis |  |  |  |  | **X** |  | **X** | **X** |  |  |  |  |  |  |  |  | **X** | **X** |  |  |  |  |

**Table 2b (Continued). Types of analyses and criteria used to define each type of analysis in internal company research reports and publications** (“modified intent to treat,” “per protocol,” “evaluable,” “efficacy evaluable,” and “week one completers”)

| **Criteria to define types of analysis** | **“Modified Intent to treat”** | | | | **“Per protocol”** | | | | **“Evaluable”** | | | | | | | | | | **“Efficacy evaluable”** | | **“Week one compl-eters”** | |
| --- | --- | --- | --- | --- | --- | --- | --- | --- | --- | --- | --- | --- | --- | --- | --- | --- | --- | --- | --- | --- | --- | --- |
|  | **945-220** | | **945-276** | | **945-291** | | **945-271** | | **879-201** | | **945-209** | | **945-210** | | **945-306** | | **945-411** | | **945-220** | | **945-209** | |
|  | Research report | Publication^4^ | Research report | Publication^8^ | Research report | Publication^6^ | Research report | Publication^7^ | Research report^a^ | Publication^10^ | Research report | Publication^5^ | Research report | Publication^1^ | Research report | Publication^3^ | Research report | Publication^9^ | Research report | Publication^4^ | Research report | Publication^5^ |
| Meet cut-off criteria for baseline variables such as age, CGIS, HAM-D, YMRS, or symptom score |  |  |  |  | **X** |  |  |  |  |  |  |  |  |  |  |  |  |  |  |  |  |  |
| No protocol violations or variations |  |  |  |  |  |  | **X** | **X** |  |  |  |  |  |  |  |  | **X^c^** | **X^d^** |  |  |  |  |
| Not randomized; treated on an open label basis (“Breaking code”) |  |  |  |  |  |  |  |  | **X** |  |  |  | **X** |  |  |  |  |  |  |  | **X** |  |

**Footnotes for Table 2b**

a The Methods and Results section in the synopsis of the research report for Study 945-411 described a modified intent to treat analysis (MITT) as follows: “The MITT population defined as patients of ITT which fulfill the following criteria: Received at least 4 weeks of treatment with study drug and have the diary data for this period; have sufficient baseline data; and exclude the patients with mention of “lack of compliance” or “protocol violators” or “withdrawn for other reason than adverse event”.” The main body of the research report did not specify a MITT analysis.

b This analysis was termed in different ways throughout the research report. For example, the description on Page 17 refers to this analysis as “Evaluable” in one location and an “efficacy analyses” at another location.

c “Patients with mention of “Protocol Violators” as determined by Pfizer Study Group will not be included;” “Patients who are incorrectly randomized will not be included;” “Patients whose status is completed, withdrawn due to Lack (sic) of efficacy, or adverse event. Patients who are withdrawn for other reasons will not be included.”

d “Protocol violators and subjects who dropped out of the study due to lack of compliance, randomization errors, or for reasons other than lack of efficacy or AEs (sic) were not included in the evaluable population.”

**Table 3. Comparison of internal company research reports and publications for description of criteria used to determine inclusion of trial participants in safety analysis**

| **Criteria to define safety analysis** | **Safety analysis^a^** | | | | | | | | | | | | | | | | | | | |
| --- | --- | --- | --- | --- | --- | --- | --- | --- | --- | --- | --- | --- | --- | --- | --- | --- | --- | --- | --- | --- |
|  | **879-201** | | **945-220** | | **945-209** | | **945-291** | | **945-210** | | **945-224** | | **945-271** | | **945-276** | | **945-306** | | **945-411** | |
|  | Research report | Publication^10^ | Research report | Publication^4^ | Research report | Publication^5^ | Research report | Publication^6^ | Research report | Publication^1^ | Research report | Publication^2^ | Research report | Publication^7^ | Research report | Publication^8^ | Research report | Publication^3^ | Research report | Publication^9^ |
| Did not explicitly specify or describe criteria for safety analysis |  | **X** |  | **X** | **X** | **X** |  | **X** |  |  |  | **X** |  |  |  |  |  | **X** |  |  |
| All patient data, “regardless of length of treatment and whether or not randomized.” | **X** |  |  |  |  |  |  |  |  |  |  |  |  |  |  |  |  |  |  |  |
| All patients randomized |  |  |  |  |  |  |  |  | **X^b^** | **X** |  |  | **X** | **X** | **X** |  | **X** |  |  |  |
| Completed treatment at minimum dose and/or for a minimum duration |  |  | **X** |  |  |  | **X** |  |  |  | **X** |  | **X** | **X** | **X** | **X** | **X** |  | **X^c^** | **X** |
| Data available for outcome variable at baseline or screening |  |  |  |  |  |  |  |  |  |  |  |  |  |  |  |  |  |  |  |  |
| Data available after randomization for a specified or unspecified number of visits/days |  |  | **X** |  |  |  |  |  |  |  |  |  |  |  |  |  |  |  |  |  |
| No protocol violations or variations |  |  |  |  |  |  |  |  |  |  |  |  |  |  |  |  |  |  |  |  |

**Footnotes for Table 3**

a We did not have access to the internal company research report for Study No ID - Gorson.

b The research report included inconsistent descriptions for the type of analysis in the Results sections. We used data from Page 47 of the research report (Results section).

Methods section

Page 16 “All patients who were randomized to treatment and received study medication were evaluated for safety.”

Results section

Page 47 “Data from all randomized patients were evaluated for safety.”

c The research report included inconsistent descriptions for the type of analysis in the Results sections. We used data from Page 39 of the research report (Results section).

Methods section

Page 31 “All patients randomized to study medication were evaluated for safety.”

Page 34 “The safety population was also defined in the statistical analysis plan as all patients that receive (sic) at least one dose of drug study (sic).”

**Table 4 Verbatim text from documents we examined for trials specified in Table 2 of the article as having had a disagreement between protocol and research report on description of definition for intent to treat analysis**

| **Study ID** | **Type of document** | **Verbatim description of definition for intent to treat analysis** |
| --- | --- | --- |
| ***Protocol versus research report*** | | |
| 945-224 | Protocol | "The population analyzed will be the Intent-to-Treat one: this includes all patients randomized to treatment who received at least 1 dose of study medication." |
|  | Research report | "The primary and secondary efficacy criteria and quality of life for the double-blind phase were analyzed on the intent-to-treat (ITT) population. The ITT population was defined as all patients randomized who received at least 1 dose of study medication in the double-blind phase.” |
| 945-411 | Protocol | "The population analyzed will be intent-to-treat (ITT): this includes all patients who received at least 1 dose of study medication.” |
|  | Research report | "The intent-to-treat (ITT) population was defined as population of patients that received at least one dose of study medication and have baseline data." |

**Table 5 Verbatim text from documents we examined for trials specified in Table 2 of the article as having had a disagreement between protocol and research report on description of definition for safety analysis**

| **Study ID** | **Type of document** | **Verbatim description of definition for safety analysis** |
| --- | --- | --- |
| ***Protocol versus research report*** | | |
| 945-210 | Protocol | "All patients who have taken study medication will be included in the evaluation of safety data." |
|  | Research report^a^ | “Data from all randomized patients were evaluated for safety.” |
| 945-306 | Protocol | “All patients who have taken study medication will be included in the evaluation of safety data.” |
|  | Research report | "All 305 patients who were randomised into the study and took at least one dose of study drug are included in the safety evaluable population." |

**Footnotes for Table 5**

a The research report included inconsistent descriptions for the type of analysis in the Results sections. We used data from Page 47 of the research report (Results section).

Methods section

Page 16 “All patients who were randomized to treatment and received study medication were evaluated for safety.”

Results section

Page 47 “Data from all randomized patients were evaluated for safety.”

**Table 6 Verbatim text from documents we examined for trials specified in Table 2 of the article as having had a disagreement between protocol and publication on description of definition for intent to treat analysis**

| **Study ID** | **Type of document** | **Verbatim description of definition for intent to treat analysis** |
| --- | --- | --- |
| ***Protocol versus publication*** | | |
| 945-209 | Protocol | “A secondary population will be the Intent-to-Treat population which is defined as all patients randomized to treatment and who have at least 1 postrandomization visit." |
|  | Publication^5^ | "The efficacy analyses were carried out on the intent-to-treat (ITT) population that included all randomized patients who received at least one dose of study medication.” |
| 945-411 | Protocol | "The population analyzed will be intent-to-treat (ITT): this includes all patients who received at least 1 dose of study medication.” |
|  | Publication^9^ | "Efficacy analyses (except responder rate) were conducted on an ITT population consisting of all randomised subjects who received at least one dose of study medication and had baseline data." |

**Table 7 Verbatim text from documents we examined for trials specified in Table 2 of the article as having had a disagreement between protocol and publication on description of definition for safety analysis**

| **Study ID** | **Type of document** | **Verbatim description of definition for safety analysis** |
| --- | --- | --- |
| ***Protocol versus publication*** | | |
| 945-210 | Protocol | "All patients who have taken study medication will be included in the evaluation of safety data." |
|  | Publication^1^ | "All patients randomized to treatment were evaluated for safety." |

**Table 8 Verbatim text from documents we examined for trials specified in Table 2 of the article as having had a disagreement between research report and publication on description of definition for intent to treat analysis**

| **Study ID** | **Type of document** | **Verbatim description of definition for Intent to treat analysis** |
| --- | --- | --- |
| ***Research report versus publication*** | | |
| 945-276 | Research report | "A primary efficacy analysis has been planned in the protocol on valid Intent-to-treat (ITT) population."  "Patients evaluable for the primary efficacy ITT analysis must have at least 3 days of evaluation on diary and have taken least one dose of study drug." |
|  | Publication^8^ | "The main analysis was performed on the intent-to-treat (ITT) population (all patients who received at least one study medication), imputing missing longitudinal data with the average of the observed data." [Omitted citation to reference in original text]. |
| 945-411 | Research report | "The intent-to-treat (ITT) population was defined as population of patients that received at least one dose of study medication and have baseline data." |
|  | Publication^9^ | "Efficacy analyses (except responder rate) were conducted on an ITT population consisting of all randomised subjects who received at least one dose of study medication and had baseline data." |

**Table 9 Verbatim text from documents we examined for trials specified in Table 2 of the article as having had a disagreement between research report and publication on description of definition for safety analysis**

| **Study ID** | **Type of document** | **Verbatim description of definition for safety analysis** |
| --- | --- | --- |
| ***Research report versus publication*** | | |
| 945-276 | Research report | "All patients randomized who took at least one dose of study drug have been evaluated for safety analysis." |
|  | Publication^8^ | "Safety data analysis was performed on the ITT population, and frequency distributions were used to present the results.”  "The main analysis was performed on the intent-to-treat (ITT) population (all patients who received at least one study medication), imputing missing longitudinal data with the average of the observed data." [Omitted citation to reference in original text]. |

**References**

1. Backonja M, Beydoun A, Edwards KR, et al for the Gabapentin Diabetic Neuropathy Study Group. Gabapentin for the symptomatic treatment of painful neuropathy in patients with diabetes mellitus. A randomized controlled trial. JAMA 1998; 280 (21): 1831 - 6.
2. Backonja M, Glanzman RL. Gabapentin dosing for neuropathic pain: evidence from randomized, placebo-controlled clinical trials. Clin Ther 2003; 25 (1): 81 - 104.
3. Serpell MG, Neuropathic Pain Study Group. Gabapentin in neuropathic pain syndromes: a randomised, double-blind, placebo-controlled trial. Pain. 2002; 99:557 - 66.
4. Mathew NT, Rapoport A, Saper J, et al. Efficacy of gabapentin in migraine prophylaxis. Headache 2001; 41: 119 - 28.
5. Pande AC, Crockatt JG, Janney CA, Werth JL, Tsaroucha G., Gabapentin Bipolar Disorder Study Group. Gabapentin in bipolar disorder: a placebo-controlled trial of adjunctive therapy. Bipolar Disord 2000; 2: 249 - 55.
6. Vieta E, Goikolea JM, Martinez-Aran A, et al. A double-blind, randomized, placebo-controlled, prophylaxis study of adjunctive gabapentin for bipolar disorder. J Clin Psychiatry 2006; 67(3): 473 - 7.
7. Gordh TE, Stubhaug A, Jensen TS, et al. Gabapentin in traumatic nerve injury pain: A randomized, double-blind, placebo-controlled, cross-over, multi-center study. Pain 2008; 138: 255 - 66.
8. Caraceni A, Zecca E, Bonezzi C, et al. Gabapentin for neuropathic cancer pain: a randomised controlled trial from the Gabapentin Cancer Pain Study Group. J Clin Oncol 2004; 22(14): 2909 - 17.
9. Gomez-Perez FJ, Perez-Monteverde A, Nascimento O, et al for the Latin American Diabetic Neuropathy Study Group. Gabapentin for the treatment of painful diabetic neuropathy: dosing to achieve optimal clinical response. Br J Diabetes Vasc Dis 2004; 4(3): 173 - 8.
10. Wessely P, Baumgartner Ch, Klingler D, et al. Preliminary results of a double-blind study with the new migraine prophylactic drug gabapentin. Cephalalgia 1987; 7 (Supplement 6): 477 - 8.
11. Gorson KC, Schott C, Herman R, Ropper AH, Rand WM (1999) Gabapentin in the treatment of painful diabetic neuropathy: a placebo controlled, double blind, crossover trial. J Neurol Neurosurg Psychiatry 66: 251-252.
